# Patent Foramen Ovale Closure Alleviates Long-Term Drug-Refractory Epilepsy: A Case Report

**DOI:** 10.3390/jcm15207906

**Published:** 2026-10-13

**Authors:** Yaning Sun, Xianying Zhao, Xuehua Cui, Xiaolei Song, Zihao Wang, Wenbo Liu, Zhengyang Zhang, Wenling Li

**Affiliations:** 1 Department of Neurosurgery, Hebei Province of Children’s Hospital, Shijiazhuang 050031, China; 2 Department of Neurosurgery, The Second Hospital of Hebei Medical University, Shijiazhuang 050061, China

**Keywords:** drug-refractory epilepsy, patent foramen ovale, percutaneous closure, electroencephalography

## Abstract

**Background:** Drug-refractory epilepsy (DRE) poses a persistent clinical burden, and the identification of reversible extracranial contributing factors represents a promising strategy to improve seizure control. Patent foramen ovale (PFO) with right-to-left shunt (RLS) has been recognized as a potential modifiable driver of epileptic seizures, yet clinical data on patients with extremely long-standing DRE exceeding 40 years remain exceedingly scarce. **Methods:** We report a 46-year-old female patient with a 44-year history of DRE, who experienced daily secondarily generalized tonic–clonic seizures (GTCSs) despite sequential treatment with six mechanistically distinct antiseizure medications. Systematic workup was performed and the patient underwent percutaneous transcatheter PFO closure, with a follow-up period of 14 months. **Results:** Systematic workup confirmed severe PFO-related RLS (grade III by contrast transthoracic echocardiography, grade IV by contrast transcranial Doppler), while brain MRI revealed no definite structural epileptogenic lesions other than an old ischemic infarct in the right corona radiata. At the latest follow-up, the patient had achieved complete freedom from GTCS with a markedly reduced antiseizure medication burden, retaining only infrequent mild focal absence-like seizures. Follow-up video electroencephalography unmasked previously occult focal epileptiform discharges in the left temporal region. **Conclusions:** This case suggests that severe PFO-related RLS may serve as a reversible aggravating factor even in decades-long refractory epilepsy, and provides electroclinical evidence supporting a hypothetical two-hit model of seizure generalization. PFO screening may be considered in selected patients with cryptogenic drug-refractory epilepsy, particularly those with non-lesional MRI, non-localizing EEG, and morning-predominant seizures.

## 1. Introduction

Epilepsy is one of the most prevalent chronic neurological disorders worldwide, affecting approximately 70 million people globally [1]. Despite continuous advances in antiseizure medications (ASMs), around 30% of patients with epilepsy eventually develop drug-refractory epilepsy (DRE), which is defined as failure of two or more tolerated, appropriately selected and dosed ASM regimens to achieve sustained seizure freedom [1,2,3]. The etiology of DRE is highly heterogeneous, covering structural, genetic, infectious, metabolic and idiopathic causes, and a considerable proportion of cases remain cryptogenic even after comprehensive intracranial evaluation [4]. Beyond recurrent seizures themselves, long-standing DRE imposes multifaceted and profound burdens: it is associated with elevated risks of cognitive decline, psychiatric comorbidities, accidental injury and sudden unexpected death in epilepsy (SUDEP); meanwhile, long-term medication, repeated hospital visits and disability caused by seizures bring heavy healthcare expenditure, lost productivity, and tremendous psychological and economic pressure on patients and their families [1,2]. For patients with cryptogenic DRE, conventional diagnostic workup predominantly focuses on intracranial pathologies and often overlooks extracranial, reversible contributing factors. Identifying such modifiable extracranial triggers has therefore become a key clinical priority, as targeted intervention may substantially alter disease trajectory and reduce disease burden even without eliminating the primary epileptogenic lesion [5].

As one of the most common congenital intracardiac shunt disorders, patent foramen ovale (PFO) represents a relatively underrecognized modifiable factor in clinical practice, as supported by prior cohort studies. PFO is present in approximately 25% of the general adult population [6], characterized by incomplete fusion of the atrial septum primum and secundum with potential right-to-left shunt (RLS). In recent decades, accumulating clinical observations and cohort studies have revealed a robust association between PFO-related RLS and epileptic seizures [7,8,9,10]. Proposed pathophysiological mechanisms include chronic cerebral microembolism, intermittent cerebral hypoxemia, and direct shunting of proinflammatory mediators into the cerebral circulation, all of which can elevate cortical excitability and lower the threshold for seizure generalization [10,11]. Nevertheless, the causal relationship between PFO and epilepsy has not been fully established, and PFO screening is still rarely incorporated into the routine diagnostic workup for epilepsy [9,10,11]. As a result, PFO-related seizures are frequently misdiagnosed as idiopathic epilepsy, subjecting patients to years or even decades of ineffective polypharmacy, unnecessary adverse drug reactions, persistent seizure deterioration, and mounting economic and psychological strain on both patients and their families [10,11,12].

Notably, most existing studies and case reports focus on patients with relatively short disease duration, and clinical data on DRE with a disease course exceeding 40 years are extremely scarce. Furthermore, the dynamic electroclinical changes after PFO closure in long-standing refractory epilepsy remain poorly characterized [13]. In this report, we present a case of 44-year drug-refractory epilepsy with severe PFO-related RLS, in which percutaneous PFO closure achieved complete resolution of generalized tonic–clonic seizures and a significant reduction in ASM burden, while previously occult focal epileptiform discharges were unmasked on follow-up electroencephalography. This case suggests that PFO-related RLS may serve as a reversible aggravating factor in long-standing cryptogenic DRE and underscores the clinical value of cardiac shunt screening in selected patients with treatment-resistant epilepsy of unknown cause.

## 2. Case Description

A 46-year-old female patient presented to our epilepsy center on 11 March 2025, for evaluation and management of recurrent drug-refractory epileptic seizures.

### 2.1. Clinical History

The patient’s first seizure occurred at the age of 2 years (44 years prior to admission), with no identifiable precipitating cause. The initial seizures presented as loss of consciousness, generalized tonic–clonic convulsions, and conjugate deviation of the head and eyes to the right, each episode lasting 5 to 10 min before spontaneous resolution. The seizure frequency was 1 to 2 episodes every 3 to 4 days, and no medical treatment was initiated at that time, and the patient did not receive formal antiseizure medication therapy until adulthood.

As the disease progressed, the patient developed absence-like seizures that secondarily progressed to generalized tonic–clonic seizures (GTCSs), maintaining a frequency of 1 to 2 episodes every 3 to 4 days. Seizures predominantly occurred during the transition from sleep to wakefulness in the early morning. She was prescribed levetiracetam 1.25 g twice daily and phenytoin sodium 0.1 g three times daily. Levetiracetam had been initiated approximately 10 years prior to admission, and the patient reported good adherence to all prescribed regimens; however, no satisfactory seizure control was achieved, with persistent intermittent attacks.

In the period immediately before admission, her seizure frequency worsened to 2 to 3 episodes per day, all characterized by absence-like seizures with secondary GTCS. At a local hospital, her regimen was escalated to include carbamazepine 0.2 g three times daily, sodium valproate sustained-release tablets 0.5 g three times daily, and clonazepam 2 mg once nightly. Despite this five-drug polytherapy, daily seizures persisted without improvement.

Upon admission to our department, the patient continued to experience frequent daily seizures. Lacosamide 100 mg twice daily was added to her regimen, but seizure control remained unachieved. During hospitalization, she presented with an episode of status epilepticus: involuntary tremor of the left upper limb occurred first, followed by generalized limb rigidity, trismus, loss of consciousness, and tachypnea after 1 min. Intravenous injection of diazepam 10 mg failed to terminate the seizure; subsequent intramuscular injection of midazolam 10 mg achieved gradual relief of muscle tone and restoration of normal oxygen saturation after 10 min. This episode was directly observed and managed by the inpatient clinical team and fulfilled the ILAE clinical criteria for status epilepticus (a generalized convulsive seizure lasting more than 5 min) (Table 1).

Past Medical History: The patient had a history of high fever up to 40.0 °C in early childhood, without febrile convulsions. She had a 2-year history of hypertension, with a maximum blood pressure of 150/95 mmHg, treated with irregular telmisartan. She had undergone appendectomy at the age of 36 and hysteromyomectomy at the age of 45. There was no history of coronary heart disease, diabetes mellitus, head trauma, blood transfusion, or known drug allergy.

Neurological Examination: On admission, the patient was fully conscious with normal cognitive response. Cranial appearance was normal with normal head circumference. Muscle strength and muscle tone of all four limbs were within normal limits, and pathological reflexes were bilaterally negative.

### 2.2. Auxiliary Examinations

**Figure 1:**
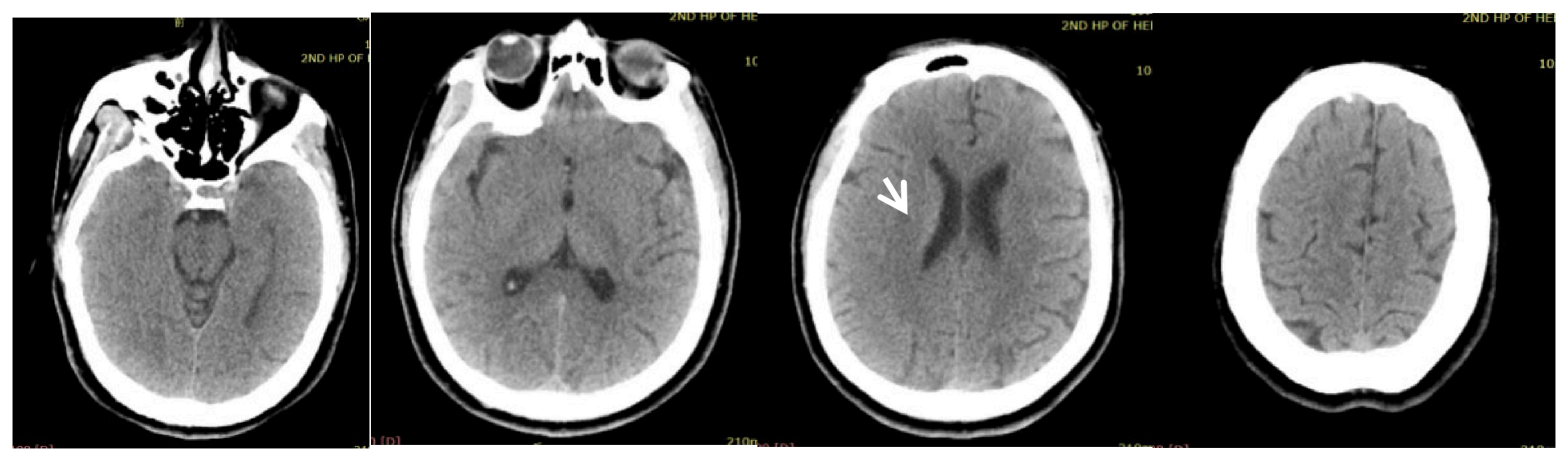
Shows cranial CT acquired on 11 March 2025. A small patchy hypodense shadow was suspected in the right corona radiata, highly suggestive of an ischemic lesion, with no other intracranial abnormalities detected. Arrow indicates the suspected ischemic lesion in the right corona radiata.

**Figure 2:**
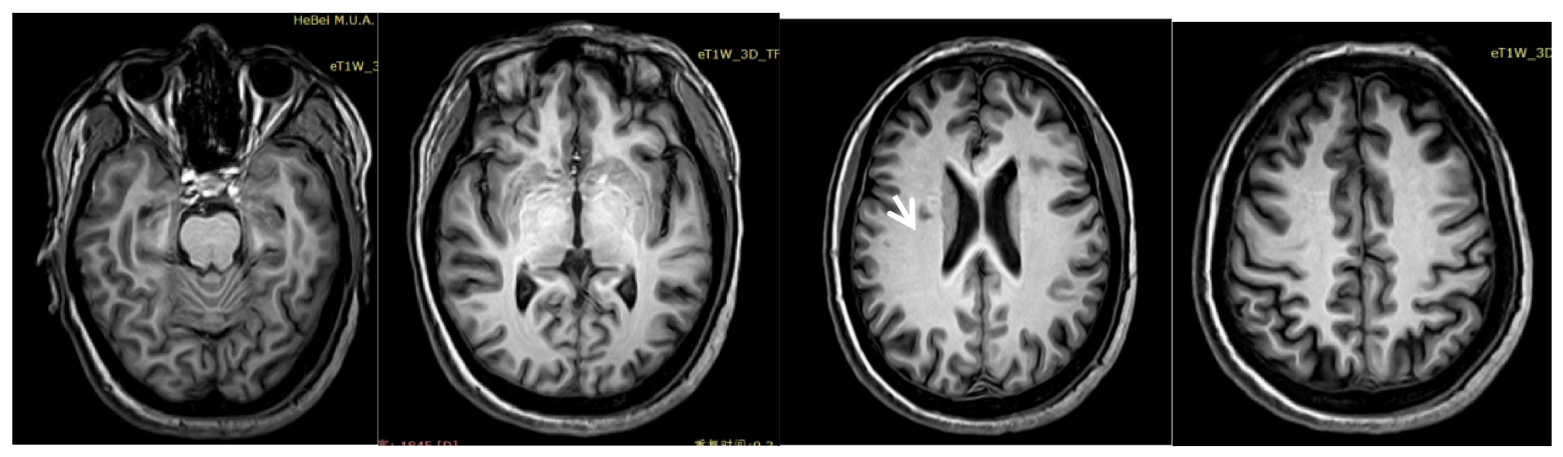
13 March 2025. Brain MRI with dedicated epilepsy protocol demonstrated symmetric hippocampi with normal signal intensity bilaterally. An established ischemic infarct was visualized within the right corona radiata, and no additional structural epileptogenic lesions were observed. Arrow indicates the old ischemic infarct in the right corona radiata.

Neuroimaging: Cranial computed tomography (CT) performed on 11 March 2025 revealed a suspected small hypodense lesion in the right corona radiata, suggestive of an ischemic focus, with no other abnormalities (Figure 1). Brain magnetic resonance imaging (MRI) with dedicated epilepsy protocol on 13 March 2025 showed symmetric bilateral hippocampi with normal signal intensity, and an old ischemic infarct in the right corona radiata. No other structural epileptogenic lesions were identified (Figure 2).

**Figure 3:**
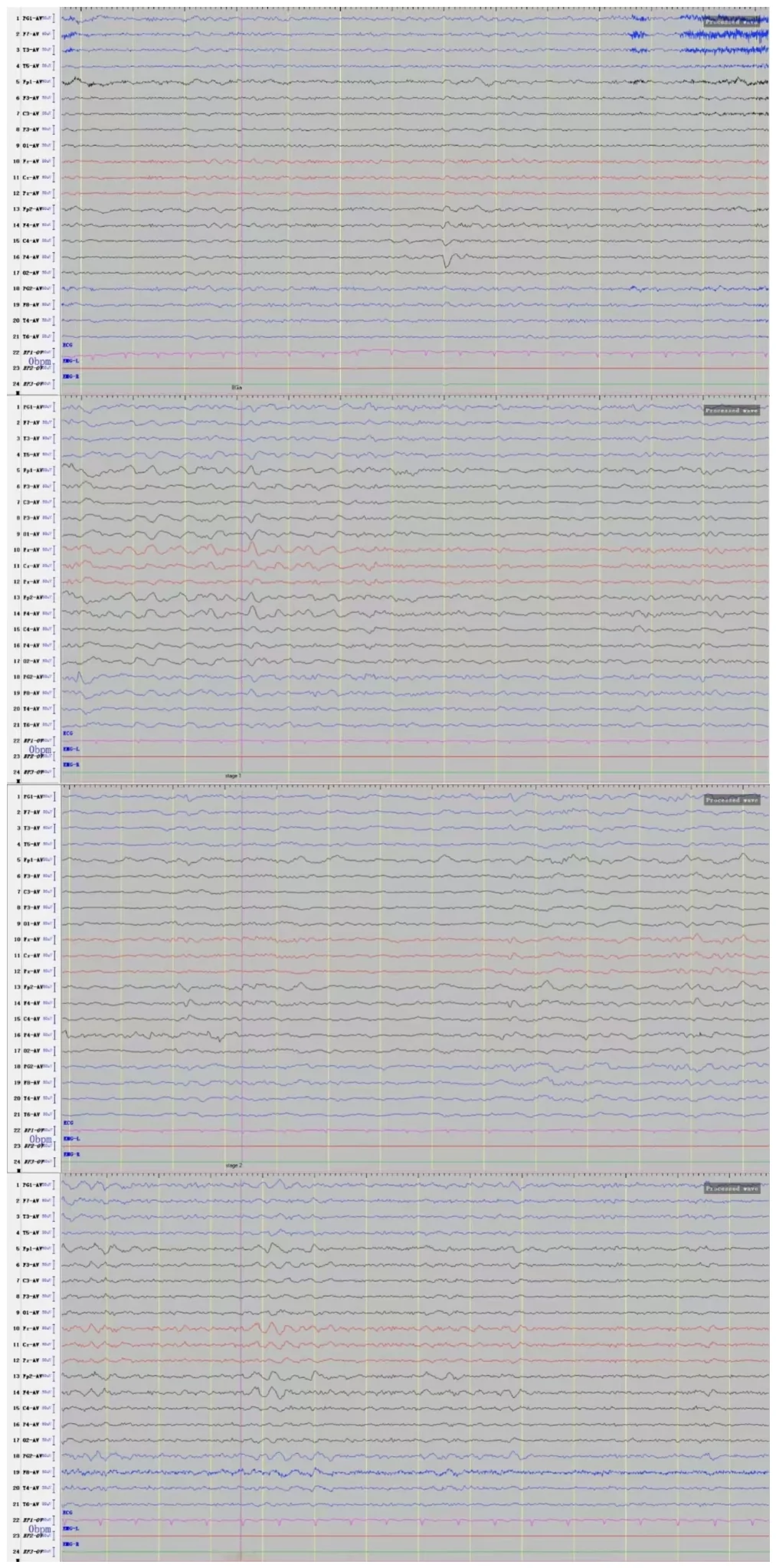
A 16 h inpatient VEEG was performed on 11 March 2025. One clinical event characterized by generalized muscle tension was captured during the sleep–wake transition period; synchronous EEG traces displayed diffuse myogenic artifacts across all channels, without identifiable epileptiform discharges or typical ictal electrographic evolution, and the episode lasted for 4 min, which was classified as a non-epileptic event. Interictal recordings revealed diffuse slow waves predominantly distributed over bilateral frontal, midline and paramidline leads (Fz, Cz, Fp2, F3, Fp1, C4-C3), with scattered low-amplitude spike-and-wave complexes more prominent over the right cerebral hemisphere. Arrows in the figure mark the interictal spike-and-wave complexes. Because the captured clinical event was non-epileptic with diffuse myogenic artifacts, no typical ictal electrographic transition from normal background to seizure discharge was present to display.

Electrophysiology: Sixteen-hour video electroencephalography (VEEG) performed on 11 March 2025 recorded an episode of generalized muscle tension upon sleep arousal. Concurrent EEG showed diffuse myogenic artifacts across all leads, without identifiable epileptiform discharges or electrographic evolution, and the episode lasted 4 min, interpreted as a non-epileptic event. Interictally, slow waves were observed in bilateral frontal regions, midline regions, and paramidline regions (Fz, Cz, Fp2, F3, Fp1, C4-C3), with scattered low-amplitude spike-and-slow waves, more prominent on the right side. No clear focal epileptogenic zone was localized (Figure 3). Of note, this event was classified as non-epileptic on both clinical and electrographic grounds, and no typical ictal epileptiform discharges were captured during the recording. In this report, the term “absence-like seizures” is used as a clinical description of the semiology of the patient’s focal seizures and does not imply electrographically confirmed absence seizures.

Given the highly stereotyped seizure semiology and documented status epilepticus, clinical suspicion for epilepsy remained high. Psychogenic non-epileptic seizures (PNES) were considered in the differential diagnosis; however, the highly stereotyped semiology with secondary generalization, the documented episode of status epilepticus, the interictal epileptiform discharges, and the seizure predilection for sleep–wake transitions were not typical of PNES, supporting a clinical diagnosis of epilepsy according to ILAE criteria. However, the absence of definitive focal epileptiform discharges on VEEG, combined with unremarkable structural neuroimaging and refractoriness to multiple antiseizure medications, prompted us to broaden the differential diagnosis. We hypothesized that an extracranial reversible factor might be driving seizure exacerbation, and cardiac right-to-left shunt secondary to patent foramen ovale was included in the diagnostic workup. Further cardiac evaluations were therefore arranged.

Laboratory Tests: Cerebrospinal fluid (CSF) examination on 17 March 2025 showed colorless and transparent fluid, with a total cell count of 1 × 10^6^/L and a white blood cell count of 1 × 10^6^/L. CSF biochemistry showed protein 0.29 g/L and glucose 3.41 mmol/L, both within normal ranges. Endocrine testing revealed elevated parathyroid hormone (76.20 pg/mL, reference range 12–65 pg/mL) and slightly elevated prolactin (27.73 ng/mL, reference range 2.74–19.64 ng/mL). Thyroid function and other pituitary hormones (LH, FSH, GH, ACTH, TSH) were within normal limits. Routine blood tests showed no significant abnormalities.

**Figure 4:**
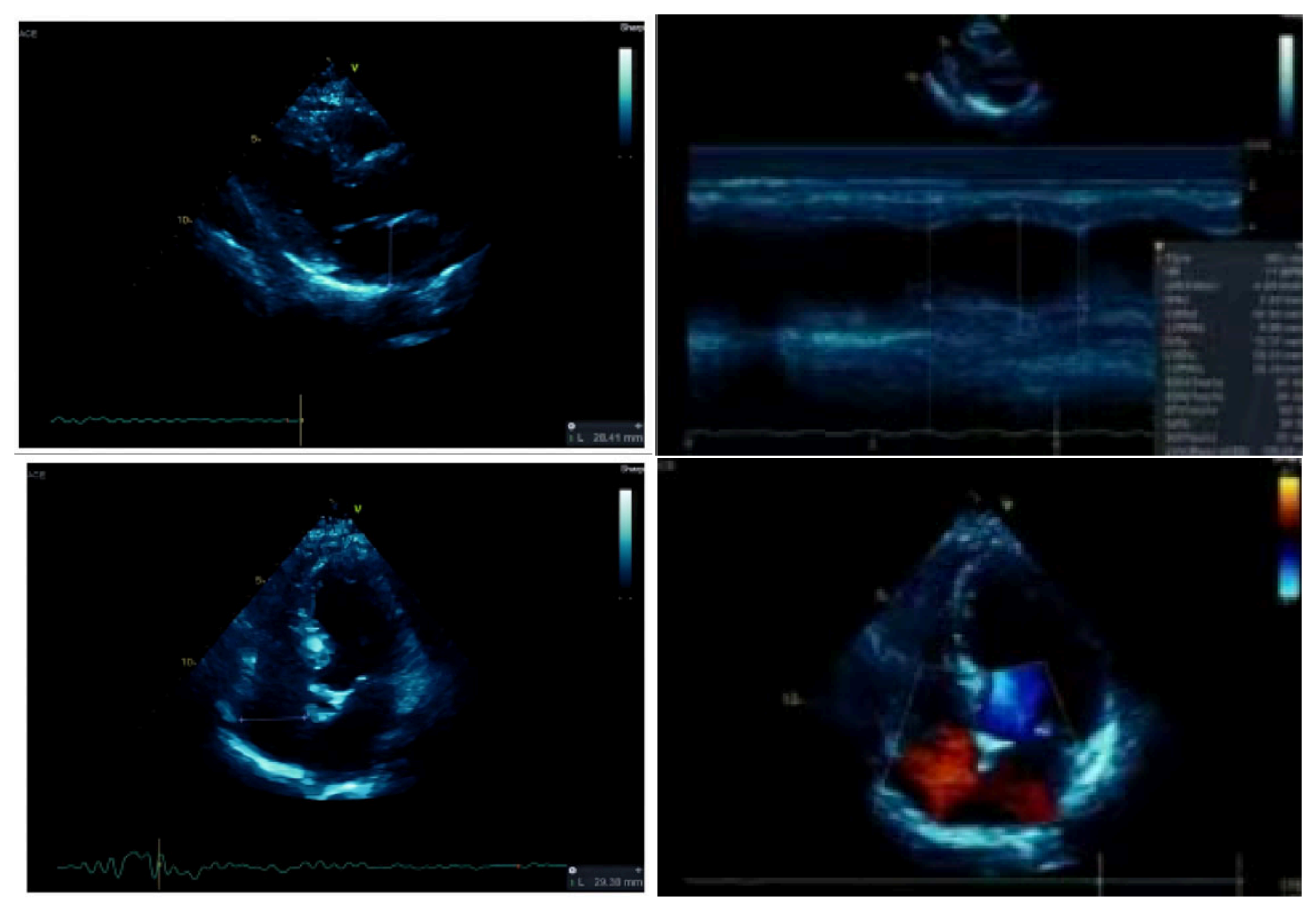
Transthoracic echocardiography (TTE) was conducted on 17 March 2025. The left ventricular ejection fraction (LVEF) measured by the Simpson method was 62.6%. Color Doppler flow imaging (CDFI) detected mild diastolic aortic regurgitation and mild systolic mitral and tricuspid regurgitation. Tissue Doppler imaging yielded an e’ velocity of 6 cm at the mitral annular septal side and an E/e’ ratio of 11.1, consistent with grade I left ventricular diastolic dysfunction, while left ventricular filling pressure remained normal. No other structural cardiac abnormalities were noted. The final diagnosis was mild aortic, mitral and tricuspid regurgitation.

Cardiac Assessment: Physical examination revealed a regular heart rate and rhythm, no pathological murmurs on cardiac auscultation, and no cyanosis, clubbing, or peripheral edema. Resting oxygen saturation was normal (96–98% on room air); oxygen saturation was not routinely monitored during seizures. Transthoracic echocardiography on 17 March 2025 showed mild aortic regurgitation, mild mitral and tricuspid regurgitation, and grade I left ventricular diastolic dysfunction, with a normal left ventricular ejection fraction of 62.6% (Figure 4). No right-to-left shunt was visible on routine color Doppler imaging at rest; the shunt was detected only on contrast-enhanced examinations with the Valsalva maneuver.

**Figure 5:**
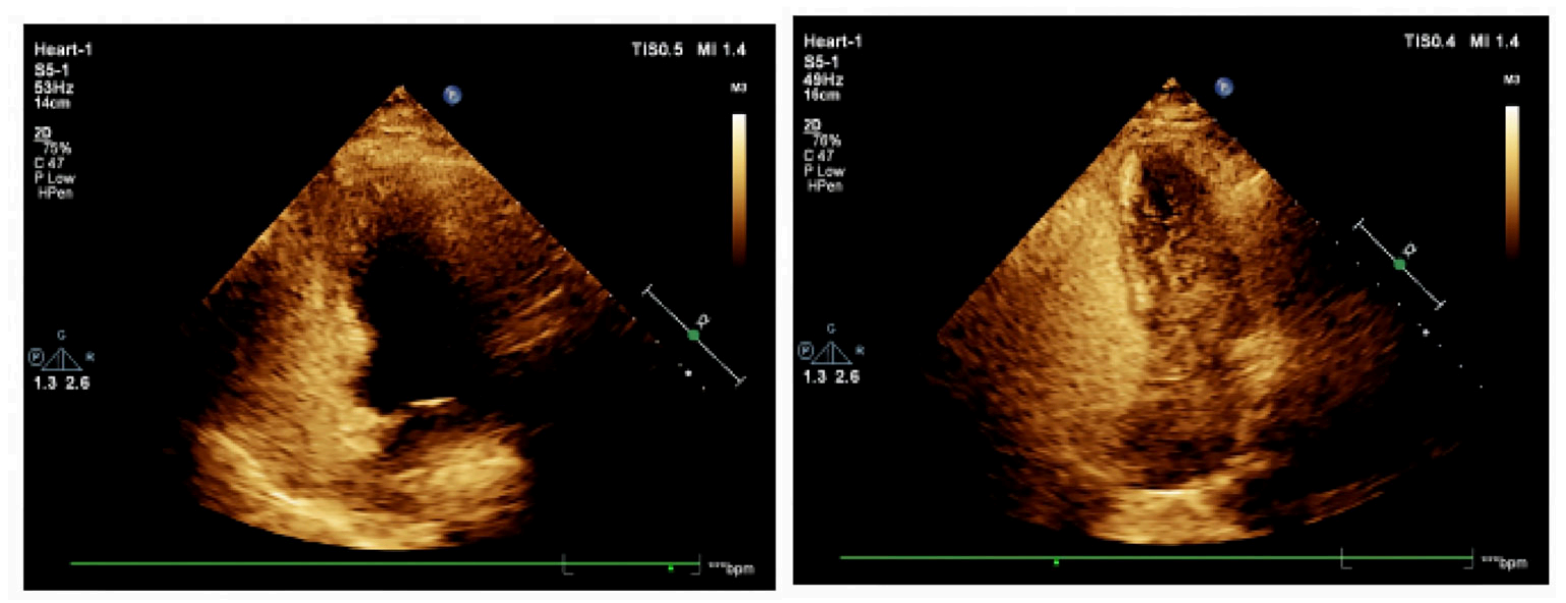
Contrast transthoracic echocardiography (cTTE, 19 March 2025) yielded positive results with grade III right-to-left shunt (RLS).

**Figure 6:**
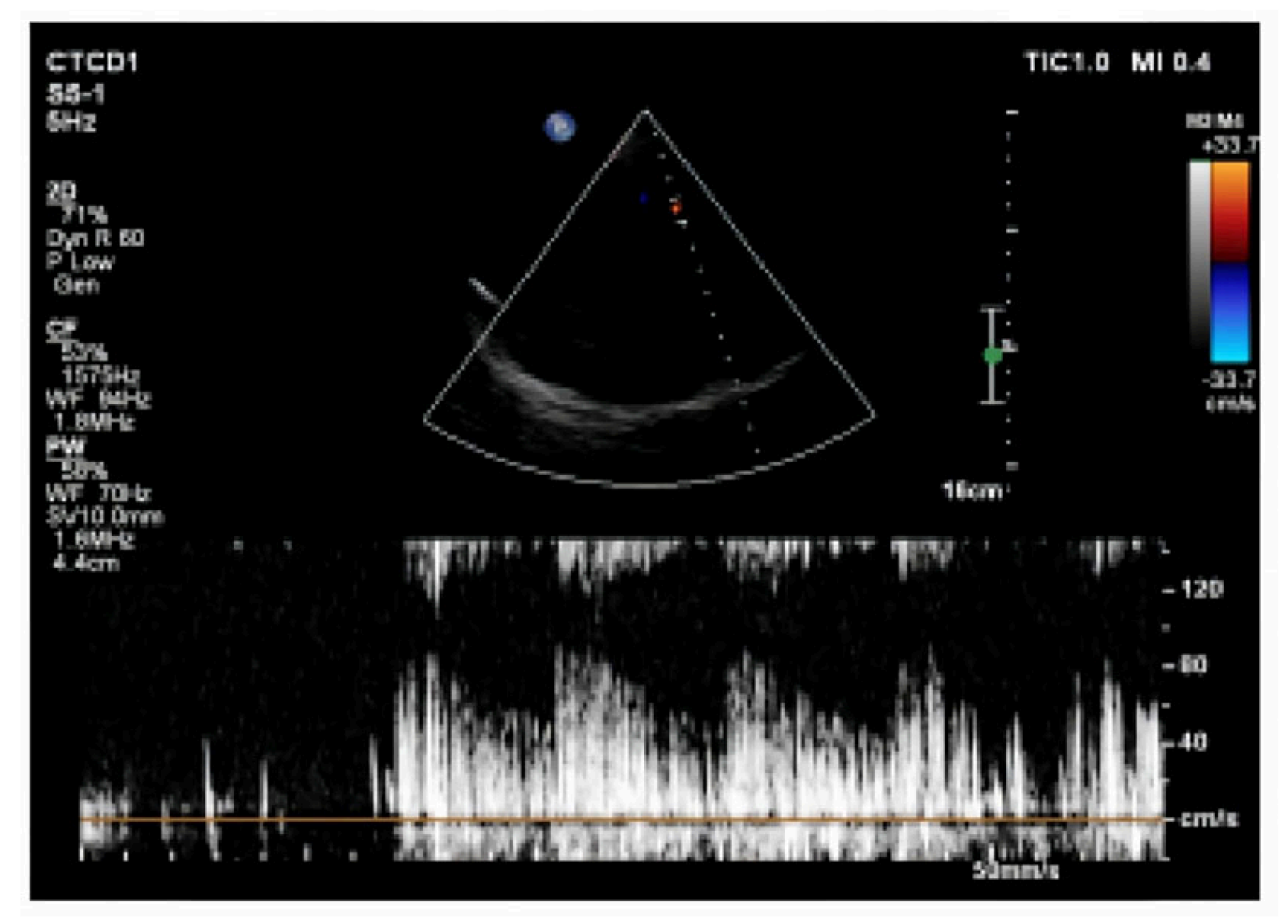
Contrast transcranial Doppler (cTCD) foam test (19 March 2025, supine position): No microembolic signals were detected in the right middle cerebral artery (MCA) under resting respiration. After standardized Valsalva maneuver, massive microembolic signals appeared in the right MCA at the 2nd second, presenting a shower-like pattern, corresponding to grade IV RLS. The cTCD result was positive (grade IV shunt).

Contrast transthoracic echocardiography (cTTE) on 19 March 2025 demonstrated grade III right-to-left shunt (RLS) under Valsalva maneuver, with the left heart chamber filled with microbubbles within three cardiac cycles; grade III was defined as the appearance of more than 30 microbubbles in the left heart chambers within three cardiac cycles after the Valsalva maneuver, according to standard clinical consensus criteria (Figure 5). Contrast transcranial Doppler (cTCD) foam test on the same day was positive at grade IV, defined as a “curtain/shower” pattern of microembolic signals after the Valsalva maneuver according to standard clinical consensus criteria: microembolic signals appeared in the right middle cerebral artery at the 2nd second after Valsalva maneuver, presenting as a “rain curtain” pattern (Figure 6).

## 3. Intervention and Follow-Up

After multidisciplinary discussion, patent foramen ovale with severe/significant right-to-left shunt was considered a major reversible contributing factor to the patient’s refractory seizures. Percutaneous transcatheter PFO closure was performed on 22 March 2025 via the right femoral venous approach under local anesthesia. Intraoperatively, the maximum diameter of the foramen ovale was approximately 1.0 mm, an anatomical size within the typical range for PFO, and a 35 mm occluder was successfully deployed. Intraoperative echocardiography confirmed optimal device positioning with no residual shunt and normal valvular function.

**Figure 7:**
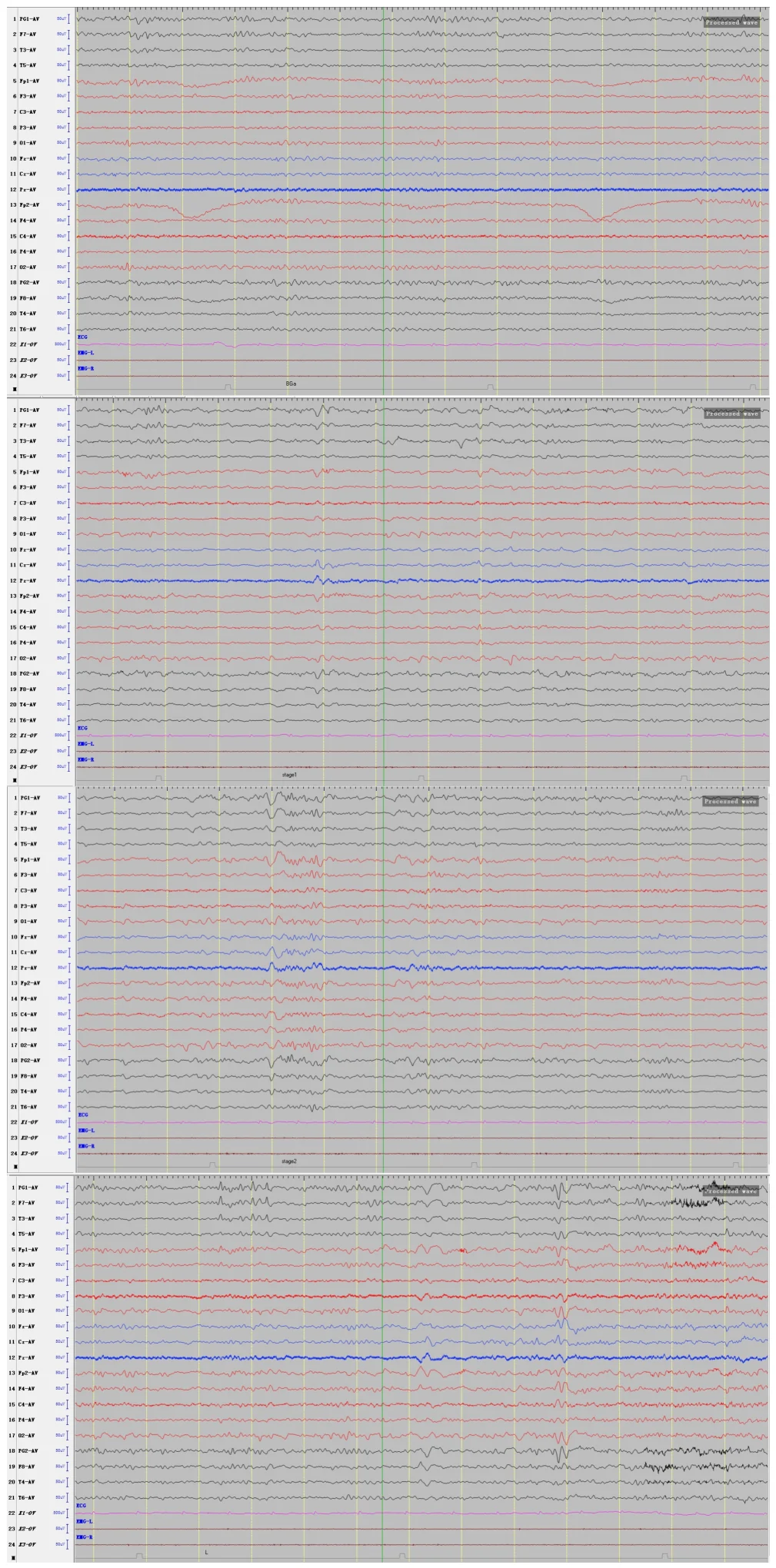
Follow-up electroencephalography performed on 31 March 2026: The dominant rhythm over the occipital lobe was a mixed 7–8 Hz low-amplitude wave with bilateral symmetry and complete suppression upon eye opening. Scattered low-amplitude fast waves and theta waves were visible across all leads. Epileptiform discharge summary: Spikes and spike-and-wave complexes originating from the left temporal region. Arrows in the figure indicate the left temporal spikes and spike-wave complexes.

At the 1-year follow-up (31 March 2026), the patient reported complete absence of GTCS since the procedure. Follow-up VEEG showed a dominant occipital rhythm of 7–8 Hz with bilateral symmetry, and focal spikes and spike-and-slow waves were detected in the left temporal region (Figure 7).

At the 14-month follow-up (May 2026), the patient remained free of GTCS, with only infrequent absence-like seizures. Her antiseizure medication regimen had been reduced to levetiracetam 0.75 g twice daily and lacosamide 150 mg twice daily, representing a significant reduction in overall medication burden.

## 4. Discussion

To the best of our knowledge, this case reports one of the longest recorded courses of cryptogenic drug-refractory epilepsy (DRE) with remarkable seizure remission after percutaneous patent foramen ovale (PFO) closure, with a 44-year disease duration far exceeding most published case series and cohort studies focusing on PFO-related epilepsy. The patient met the official 2010 ILAE diagnostic criteria for DRE after failed treatment with six distinct classes of antiseizure medications (ASMs) at adequate therapeutic doses [1,2]. Following transcatheter PFO occlusion, disabling secondary generalized tonic–clonic seizures (GTCSs) were completely eliminated, and the dosage and quantity of ASMs were markedly reduced. Notably, follow-up video electroencephalography (VEEG) revealed left temporal focal spikes and spike-wave complexes that were invisible on baseline recordings. This seemingly paradoxical electroclinical change may be interpreted in the light of domestic and international clinical and multi-omics evidence, and we propose a hypothetical two-hit model to explain this unique phenomenon, providing real-world electroclinical evidence and standardized screening recommendations for patients with cryptogenic epilepsy lacking structural intracranial lesions.

The epidemiological link between PFO and DRE has been consistently validated in domestic and global cohorts. The general adult population carries a PFO prevalence of approximately 20–25% [6], while epilepsy patients demonstrate a significantly higher detection rate. A single-center case–control study from West China Hospital enrolled 741 epilepsy patients and 800 healthy controls; PFO positivity reached 39.0% in the epilepsy group versus 24.25% in healthy volunteers. After propensity score adjustment, patients with epilepsy had a 1.71-fold higher risk of PFO, and those with DRE were more likely to present moderate or severe right-to-left shunt (RLS) [8,9]. A Chinese cross-sectional study including 182 epilepsy patients further confirmed that grade III severe RLS served as an independent risk factor for DRE, with 40.0% of severe shunt patients meeting ILAE refractory criteria compared with only 23.0% of patients without shunt [14]. A domestic narrative review summarized multi-center clinical data and pointed out that routine neurological workups predominantly focus on intracranial lesions, resulting in long-term misdiagnosis of PFO-related epilepsy as idiopathic epilepsy and years of ineffective multi-drug therapy [7]. Our patient perfectly reflects this clinical dilemma: 44 years of recurrent daily seizures, six ineffective ASM regimens, and non-localizing baseline VEEG delayed cardiac shunt screening until comprehensive etiological assessment. For diagnostic accuracy, synchronous contrast transthoracic echocardiography (cTTE) and contrast transcranial Doppler (cTCD) are recommended as the gold-standard screening strategy, as single-modality testing bears high false-negative risks [15]. Our patient underwent combined ultrasound testing and was diagnosed with grade III RLS on cTTE and grade IV RLS on cTCD, consistent with the high-risk shunt phenotype identified in domestic epilepsy cohorts [8,14].

Multiple synergistic pathological pathways may explain how high-grade RLS exacerbates epileptic excitability and accounts for the masked focal discharges observed on initial EEG. Intermittent cerebral hypoxemia induced by unoxygenated venous blood bypassing pulmonary filtration has been proposed as a core pathogenic mechanism, supported by multi-omics analysis of peripheral biomarkers [12]. During Valsalva maneuvers or sleep–wake transitions, elevated right atrial pressure enlarges the PFO channel, allowing deoxygenated blood to enter systemic circulation and trigger hypoxic brain injury—consistent with our patient’s seizure predilection for early morning awakening. Chronic recurrent cerebral hypoxia may upregulate central oxidative stress, activate microglia, and increase blood–brain barrier P-glycoprotein expression, which could impair central ASM bioavailability and contribute to drug resistance [12,16]. In addition, paradoxical microembolism passing through the atrial septum may contribute to cumulative cerebral ischemic injury over decades; the single old infarct visible on T1-weighted imaging in the right corona radiata may represent only the most clinically apparent of multiple subclinical microembolic events [17]. We acknowledge that T2-FLAIR imaging would be more sensitive for demonstrating the sequelae of multiple microemboli, and that FLAIR sequences were not included in the figures of this case report. Cortical spreading depression (CSD) triggered by microemboli and circulating inflammatory mediators may produce diffuse cortical suppression, which could conceal underlying focal epileptogenic activity on baseline VEEG [7,9].

We put forward a hypothetical two-hit pathogenic model to interpret the distinct pre- and post-EEG changes in this patient. The first hit refers to intrinsic left temporal focal cortical irritability, which may cause only mild absence-like focal seizures in the absence of diffuse cerebral excitation. The second hit is persistent severe PFO-RLS, which may induce widespread global cortical hyperexcitability and lower the threshold for secondary generalization of focal discharges, which may result in daily intractable GTCS. Long-standing diffuse hypoxic suppression may have masked temporal spike activity on baseline EEG; after PFO closure, cerebral oxygenation may recover, diffuse background slowing may resolve, and latent focal epileptiform discharges may become detectable on follow-up VEEG. This electrophysiological unmasking phenomenon has also been documented in domestic cohorts of patients with grade III RLS [14]. After eliminating the diffuse excitatory stimulus via occlusion, generalized seizures were permanently abolished, yet mild focal seizures persisted due to the intrinsic temporal epileptogenic substrate, which aligns with published domestic interventional follow-up data [8,12].

Accumulating evidence from domestic prospective trials and international retrospective cohorts suggests a therapeutic benefit of PFO closure in patients with high-grade RLS and DRE. The ongoing PFOCRE prospective randomized controlled trial led by West China Hospital was designed to generate high-level evidence for PFO occlusion as a targeted therapy for DRE with moderate-to-severe RLS, and its preliminary 28-case observational pre-data demonstrated that half of patients achieved ≥50% seizure frequency reduction at two-year follow-up, with complete remission of generalized attacks in most participants [11]. A European retrospective cohort also reported substantial seizure attenuation after PFO closure in refractory epilepsy patients [18]. A critical confounding factor is unlikely in our case: the overall ASM burden was markedly reduced after intervention, with four of the six preoperative medications discontinued and the dosages of the remaining two agents (levetiracetam and lacosamide) reduced, while seizure control continued to improve. Moreover, seizure improvement was observed shortly after the procedure and before any medication reduction, and GTCS remained completely absent after subsequent tapering. This argues against medication adjustment as the primary explanation and suggests that shunt elimination was a plausible contributor to the observed remission [12].

While a single case cannot justify routine universal screening, this case yields several clinically actionable considerations for the management of selected patients with cryptogenic DRE, supported by domestic ultrasound studies and national cardiovascular expert consensus [15]. First, clinicians may consider expanding the differential diagnosis beyond intracranial structural lesions for patients with multi-drug resistant epilepsy, non-localizing VEEG, non-lesional MRI, morning-predominant seizures, or incidental small cerebral infarcts; synchronous cTTE and cTCD screening for PFO may be considered in such selected patients. Second, non-localizing baseline VEEG cannot rule out focal epilepsy, as diffuse hypoxic suppression may mask latent epileptic foci; repeat VEEG after targeted cardiac intervention may help localize the epileptogenic zone and guide ASM tapering. Third, ultra-long disease duration may not be an absolute contraindication for PFO occlusion. Contrary to the outdated assumption that decades of epilepsy are irreversible, our 44-year case and domestic follow-up cohorts confirm that closure can eliminate disabling generalized seizures and reduce long-term polypharmacy burden. Fourth, multidisciplinary collaboration between neurology and cardiology would be helpful to standardize RLS grading, procedural indications, and post-procedure antiplatelet and seizure follow-up protocols. Prospective controlled studies are required before routine PFO screening or closure can be recommended for patients with drug-refractory epilepsy.

Several limitations must be acknowledged to maintain academic rigor: First, this is a single case report without a control group; definitive causal inference between PFO closure and seizure remission cannot be established, and spontaneous seizure fluctuation or placebo effect cannot be fully excluded. Second, follow-up duration is limited to 14 months; extended long-term monitoring (3–5 years) is required to confirm the durability of GTCS remission and stability of EEG focal discharges. Third, serial therapeutic drug monitoring was not completed to formally document target serum concentrations of all six preoperative ASMs, though all agents were prescribed standard therapeutic dosages per ILAE guidelines, satisfying the definition of true drug refractoriness. Fourth, no ictal electrographic confirmation of the patient’s habitual seizures was obtained on either baseline or follow-up VEEG, and per-ictal oxygen saturation was not systematically recorded. Fifth, T2-FLAIR sequences were not included in the presented neuroimaging, which limits the assessment of cumulative microembolic sequelae. 

## 5. Conclusions

The electroclinical evolution after PFO closure is consistent with a hypothetical two-hit model of seizure generalization, in which diffuse cortical hyperexcitability induced by chronic RLS may drive the progression from focal to generalized seizures. PFO closure supports a hypothetical two-hit model of seizure generalization, in which diffuse cortical hyperexcitability induced by chronic RLS drives the progression from focal to generalized seizures. For selected patients with unexplained refractory epilepsy, screening for PFO may identify a modifiable therapeutic target and yield meaningful clinical improvement.

## Figures and Tables

**Table 1:** Clinical course before and after PFO closure.

Time Period	Seizure Type	Seizure Frequency	ASM Regimen (Dose)	Key Findings/Events
Age 2–Early adulthood	GTCS: loss of consciousness, generalized tonic–clonic convulsions, conjugate deviation of head and eyes to the right	1–2 episodes every 3–4 days	None	No medical treatment initiated at onset
Adulthood, before admission	Absence-like focal seizures with secondary GTCS	1–2 episodes every 3–4 days	Levetiracetam 1.25 g bid; phenytoin sodium 0.1 g tid (initiated ~10 years before admission)	Seizures predominantly at sleep–wake transition in early morning; no satisfactory seizure control
Immediate pre-admission period	Absence-like focal seizures with secondary GTCS	2–3 episodes per day	Add: carbamazepine 0.2 g tid; sodium valproate (sustained-release) 0.5 g tid; clonazepam 2 mg once nightly (five-drug regimen)	Daily seizures persisted despite polytherapy
Admission (March 2025)	Daily seizures; one episode of status epilepticus	Daily	Add: lacosamide 100 mg bid (six ASMs in total)	Baseline VEEG: non-epileptic event; interictal slow waves and scattered spike-and-slow waves. MRI: old ischemic infarct in right corona radiata. cTTE grade III/cTCD grade IV RLS
PFO closure (22 March 2025)	—	—	—	35 mm occluder deployed; no residual shunt on intraoperative echocardiography
1-year follow-up (March 2026)	No GTCS; infrequent mild absence-like seizures	GTCS-free	Levetiracetam 0.75 g bid; lacosamide 150 mg bid	Follow-up VEEG: left temporal spikes and spike-and-wave complexes
14-month follow-up (May 2026)	No GTCS; infrequent mild absence-like seizures	GTCS-free	Levetiracetam 0.75 g bid; lacosamide 150 mg bid	Markedly reduced overall ASM burden (4 of 6 preoperative ASMs discontinued)

ASM, antiseizure medication; bid, twice daily; tid, three times daily; GTCS, generalized tonic–clonic seizures; RLS, right-to-left shunt; cTTE, contrast transthoracic echocardiography; cTCD, contrast transcranial Doppler; VEEG, video electroencephalography.

## Data Availability

The original contributions presented in this study are included in the article. Further inquiries can be directed to the corresponding author.
